# The *Salmonella* SPI-2 effector SseJ exhibits eukaryotic activator-dependent phospholipase A and glycerophospholipid : cholesterol acyltransferase activity

**DOI:** 10.1099/mic.0.2008/019075-0

**Published:** 2008-09

**Authors:** Nadine S. Lossi, Nathalie Rolhion, Anthony I. Magee, Cliona Boyle, David W. Holden

**Affiliations:** 1 Centre for Molecular Microbiology and Infection, Imperial College London, Armstrong Road, London SW7 2AZ, UK; 2 National Heart and Lung Institute, Imperial College London, London SW7 2AZ, UK

## Abstract

Intracellular replication of *Salmonella enterica* serovar Typhimurium within membrane-bound compartments, called *Salmonella*-containing vacuoles, depends on the activities of several effector proteins translocated by the *Salmonella* pathogenicity island 2 (SPI-2)-encoded type III secretion system. The SPI-2 effector protein SseJ shows similarity at the amino acid level to several GDSL lipases with glycerophospholipid : cholesterol acyltransferase (GCAT) activity. In this study, we show that catalytic serine-dependent phospholipase A (PLA) and GCAT activity of recombinant SseJ is potentiated by factor(s) present in HeLa cells, RAW macrophages and *Saccharomyces cerevisiae*. SseJ activity was enhanced with increasing amounts of, or preincubation with, eukaryotic cell extracts. Analysis of the activating factor(s) shows that it is soluble and heat- and protease-sensitive. We conclude that PLA and GCAT activities of SseJ are potentiated by proteinaceous eukaryotic factor(s).

## INTRODUCTION

*Salmonella enterica* serovar Typhimurium (*S*. Typhimurium) invades a variety of host cell types and replicates intracellularly within a membrane-bound compartment, the *Salmonella*-containing vacuole (SCV). Numerous *Salmonella* virulence genes are required for growth of this pathogen in mice; several of these are associated with the *Salmonella* pathogenicity island 2 (SPI-2) type III secretion system (T3SS). This is expressed upon bacterial entry into host cells and translocates a variety of effector proteins across the SCV into the host cell (Cirillo *et al.*, 1998; Waterman & Holden, 2003). A functional SPI-2 T3SS is essential for intracellular survival and systemic growth of *Salmonella* in mice (Hensel *et al.*, 1995; Ochman *et al.*, 1996). Approximately 20 SPI-2 effectors have been identified to date, but their molecular functions remain largely unknown (Haraga *et al.*, 2008; Waterman & Holden, 2003).

The effector protein SseJ is encoded outside SPI-2, but translocated via the SPI-2 T3SS (Miao & Miller, 2000). Interestingly, deletion of *sseJ* does not have a detectable effect on replication of *S*. Typhimurium in epithelial cells or macrophage-like cell lines, but results in a mild replication defect in elicited peritoneal macrophages (Ruiz-Albert *et al.*, 2002) and reduced virulence after intraperitoneal inoculation of BALB/c mice (Freeman *et al.*, 2003; Ruiz-Albert *et al.*, 2002). SseJ has been linked functionally to SifA by virtue of the phenotype of a *sifA sseJ* double mutant (Ruiz-Albert *et al.*, 2002). SifA is a SPI-2 T3SS effector required for the formation of tubules (called Sifs) that extend from SCVs in epithelial cells (Stein *et al.*, 1996). *sifA* mutants fail to make Sifs and gradually lose their vacuolar membrane (Beuzon *et al.*, 2000). However, the process of vacuolar membrane loss is significantly delayed in a *sifA sseJ* double mutant (Ohlson *et al.*, 2005; Ruiz-Albert *et al.*, 2002), suggesting that SseJ may help to destabilize the SCV membrane around the *sifA* mutant. Furthermore, deletion of *sseJ* has been shown to result in increased levels of Sifs (Birmingham *et al.*, 2005). Therefore, SseJ appears to oppose the activity of SifA.

The N-terminal domain of SseJ contains a translocation signal also found in some other SPI-2 effector proteins: SspH1, SspH2, SlrP, SifA, SifB and SseI (Miao & Miller, 2000). The C-terminal region (amino acids 140–408) is 29 % identical to several members of the GDSL lipase family, with highest similarity at the amino acid sequence level to a glycerophospholipid : cholesterol acyltransferase (GCAT) found in *Aeromonas hydrophila* (Brumlik & Buckley, 1996; Miao & Miller, 2000). Members of the GDSL family of lipases are characterized by the presence of a conserved GDSL motif and a catalytic triad (S-D-H) (Akoh *et al.*, 2004; Upton & Buckley, 1995). Mutation of residues of the catalytic triad causes loss of lipolytic and acyltransferase activity (Brumlik & Buckley, 1996). Alignment of SseJ with GCAT (Flieger *et al.*, 2002) reveals the presence of a GDSL motif as well as the conserved catalytic triad (S151, D274, H384). In agreement with these predicted catalytic residues, recent studies have shown that the virulence attenuation of Δ*sseJ* in mice cannot be rescued by expression of SseJ_S151A_, SseJ_D274N_ or SseJ_H384N_, indicating that these residues are important for function of SseJ *in vivo* (Ohlson *et al.*, 2005). *In vitro*, S151, D247 and H384 have been shown to be necessary for deacylase activity of recombinant SseJ (Ohlson *et al.*, 2005), supporting the notion that these represent a catalytic triad similar to those of other GDSL lipases (Ohlson *et al.*, 2005). Recently, SseJ has been shown to esterify cholesterol *in vitro*, in HeLa cells and macrophages (Nawabi *et al.*, 2008).

In this study, we analysed the biochemical activity of SseJ. We show that SseJ exhibits phospholipase A (PLA) and GCAT activity and that both enzymic activities require the presence of a eukaryotic activator.

## METHODS

### Bacterial strains and growth conditions.

*Escherichia coli* was grown in Luria–Bertani (LB) medium supplemented with carbenicillin (50 μg ml^−1^) when appropriate. *Saccharomyces cerevisiae* AH 109 was grown in YPD medium supplemented with 20 mg l^−1^ adenine hemisulfate (YPDA). Strains used in this study are summarized in Table 1.

### Reagents.

Lipofectamine 2000 transfection reagent was purchased from Invitrogen. 1,2-Dipalmitoylphosphatidylcholine (DPPC) and 1-monopalmitoyllysophosphatidylcholine (1-MPLPC) were purchased from Avanti Polar Lipids. Cholesterol and cholesteryl oleate, *para*-nitrophenyl butyrate (PNPB), esterase from porcine liver, phospholipase A2 (PLA2) from porcine liver and protease inhibitors (aprotinin, leupeptin, pepstatin A) were obtained from Sigma. [^3^H]cholesterol (specific activity 40 Ci mmol^−1^; 1.5 TBq mmol^−1^) was purchased from American Radiolabelled Chemicals.

### Cell culture.

HeLa (93021013) and RAW 264.7 (91962702) cells were obtained from the European Collection of Cell Cultures, Salisbury, UK, and grown in Dulbecco's modified Eagle's medium (DMEM) supplemented with 10 % fetal calf serum (FCS) and 2 mM glutamine at 37 °C in 5 % CO_2_.

### Plasmids.

Expression plasmids p*myc* : : *sseJ* and p*myc* : : *sseJ*_S151V_ have been described previously (Ruiz-Albert *et al.*, 2002). p*myc* : : *sseJ* expresses a full-length version of SseJ bearing an N-terminal fusion to the c-myc epitope tag in pRK5-*myc* (Lamarche *et al.*, 1996). p*myc* : : *sseJ*_S151V_ expresses a catalytically inactive SseJ_S151V_ bearing an N-terminal fusion to the c-myc epitope tag. pGEX4T2 : : *sseJ* was used for expression of GST–SseJ. This plasmid (kindly provided by Dr Stéphane Méresse, Centre d'Immunologie de Marseille-Luminy, France) contains *sseJ* under the control of an IPTG-inducible promoter. The plasmid pGEX4T2 : : *sseJ*_mt_ was constructed by site-directed mutagenesis of pGEX4T2 : : *sseJ*, using the primers sseJ-1 (5′-TTTTGGCGACGTCTTGTCTGACTCC-3′) and sseJ-2 (5′-CCATAAAAAACCGCTGCAGAACAGACTG-3′) and *Pfu* Ultra-high-fidelity polymerase (Stratagene). pGEX4T2 : : *sseJ*_mt_ expresses a catalytically inactive version of full-length SseJ, in which S in position 151 is replaced by V, fused to the C terminus of glutathione-*S*-transferase (GST). All constructs were verified by DNA sequencing prior to use. Plasmids used in this study are summarized in Table 1.

### Transfection.

HeLa cells (5×10^5^) were seeded in six-well plates 24 h prior to transfection with lipofectamine 2000 transfection reagent according to the manufacturer's manual (Invitrogen).

### GST protein purification.

Expression of GST fusion proteins was induced with 0.5 mM IPTG (Sigma) in *E. coli* BL21 (DE3) (Amersham Biosciences) at 25 °C for 4 h prior to collection of cell pellets by centrifugation. Cells were resuspended in 40 mM Tris, pH 7.4, containing Complete protease inhibitor cocktail (Roche) and subsequently lysed by passage through a French Press. The soluble fraction was isolated by ultracentrifugation at 130 000 ***g*** and subsequently incubated with glutathione–Sepharose beads (Amersham Biosciences) for 2 h at 4 °C. Beads were washed with PBS and 40 mM Tris, 100 mM NaCl, pH 8.0, before fusion proteins were eluted using 10 mM glutathione (Sigma), dialysed in 40 mM Tris, pH 7.4, and concentrated before use using Amicon-10 filter devices (Millipore).

### Cell extracts.

HeLa and RAW 264.7 cells were grown in 175 cm^2^ dishes to 80 % confluence, scraped into ice-cold PBS, pelleted at 200 ***g*** for 5 min and resuspended in homogenization buffer [8.5 % sucrose (w/v), 3 mM imidazole]. HeLa cells were broken by passage through a 22G needle; RAW 264.7 cells were broken by passage through a 27G needle. Postnuclear supernatant (PNS) was obtained after centrifugation at 1500 ***g*** for 5 min at 4 °C. Membranes and cytosol fractions of HeLa cells were separated by ultracentrifugation at 100 000 ***g*** for 1 h. Where indicated, HeLa cell cytosol was further treated by incubation with 250 μg trypsin ml^−1^ for 15 min at 30 °C, followed by addition of the trypsin inhibitor aprotinin. For preparation of *Sacc. cerevisiae* cell extract, *Sacc. cerevisiae* AH109 was grown overnight in YPDA medium at 30 °C, and cells were resuspended in 50 mM Na_3_PO_4_ buffer, pH 7.4, supplemented with protease inhibitors [10 μg aprotinin ml^−1^, 5 μg leupeptin ml^−1^, 1 μM pepstatin A and Complete protease inhibitor cocktail (Roche)]. Cells were broken by four passages through a French Press and the soluble cell extract was obtained after pelleting of cell debris by centrifugation at 14 000 ***g*** for 30 min. *E. coli* BL21 soluble extract was prepared from an overnight culture resuspended in 40 mM Tris, pH 7.4, containing Complete protease inhibitor cocktail (Roche) by passage through a French Press and subsequent ultracentrifugation at 130 000 ***g***.

### Deacylase assay.

The PNPB deacylase assay was carried out as described elsewhere (Bonelli & Jonas, 1989; Ohlson *et al.*, 2005). Briefly, 5 μg GST fusion protein was added to the assay reaction mix [1 ml containing 50 mM PNPB, 20 mM Tris, pH 7.4, and 3 % acetonitrile (v/v)] in a cuvette, and *A*_400_ was monitored over 30 min at 37 °C (UV-VIS spectrophotometer, Shimadzu UK). As a positive control, porcine liver esterase (Sigma) was used.

### Phospholipase assay.

DPPC liposomes and 1-MPLPC micelles were prepared in 1 ml 40 mM Tris, pH 7.4, by sonication with a probe at a concentration of 13.4 mM. GST fusion proteins were pre-incubated with cell lysate for 1 h at 37 °C (30 °C for yeast cell extract), where indicated. In general, 5 μg GST fusion protein was incubated at 37 °C at pH 7.4 for 2 h with 3.35 mM DPPC liposomes (3.35 mM 1-MPLPC micelles for lysophospholipase assay), with or without cell extract (100 μl if not otherwise stated; 7 mg protein ml^−1^) in a final volume of 200 μl. Porcine liver PLA2 was used as a positive control in 40 mM Tris, pH 7.4, supplemented with 10 mM CaCl_2_. Free fatty acids (FFA) were quantified using a NEFA-C kit (Wako) according to the manufacturer's manual. To test whether superoxide dismutase (SOD-1) activates SseJ, 5 μg GST fusion protein was pre-incubated with 20 μg bovine SOD-1 (Sigma) in 150 μl 40 mM Tris, pH 7.4, at 37 °C for 1 h before addition of DPPC at a final concentration of 3.35 mM and incubation for 2 h at 37 °C.

### Acyltransferase assay.

Liposomes of equimolar amounts of DPPC and partially ^3^H-labelled cholesterol (5 mM) were prepared in 20 mM Tris, pH 7.4, 160 mM KCl and 1.4 % (w/v) BSA by bath sonication for 30 min. Liposomes (50 μl) were incubated with 5 μg GST fusion protein and 100 μl eukaryotic cell lysate (7 mg ml^−1^) at 37 °C for 2 h in a final volume of 250 μl. The reaction was stopped by addition of 3 ml CHCl_3_ : MeOH (1 : 2, v/v). Lipids were extracted by the method of Bligh & Dyer (1959), and subsequently separated by TLC on glass-backed Silica 60 plates using petroleum ether : diethylether (8 : 2, v/v). The cholesterol ester (CE) fractions were scraped into Betamax ES liquid scintillation fluid (MP Biomedicals) and d.p.m. were measured.

### Statistical analysis.

For analysis of the significance of differences between samples, Student's *t* test was used. Differences denoted as significant in the text fall below a *P* value of 0.05.

## RESULTS

### Lack of enzymic activity of recombinant SseJ *in vitro*

**Fig. 1. f1:**
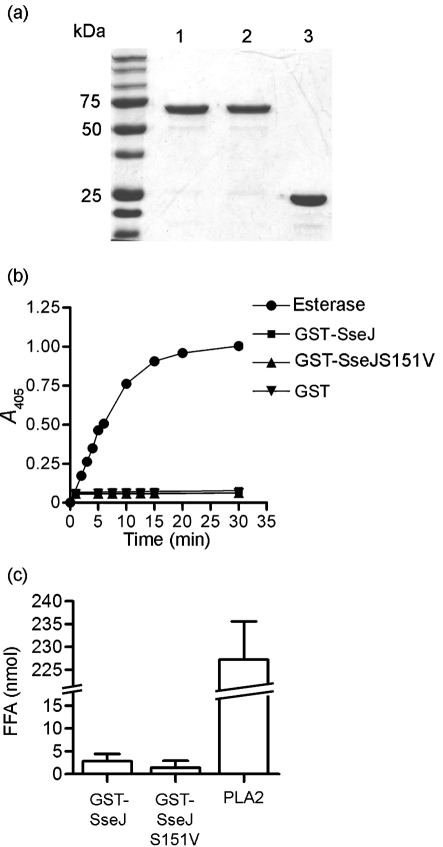
SseJ does not display esterase or phospholipase activity *in vitro*. (a) Expression and purification of SseJ and SseJS151V as GST fusion proteins. Lanes: 1, purified GST–SseJ (2 μg); 2, purified GST–SseJS151V (2 μg); 3, purified GST (2 μg). (b) GST–SseJ, GST–SseJS151V or GST was incubated with PNPB at 37 °C, pH 7.4, while *A*_405_ was monitored over 30 min. Porcine liver esterase was used as a positive control. GST and GST–SseJS151V were used as negative controls. These data represent the mean from two independent experiments performed in triplicate. (c) GST–SseJ, GST–SseJS151V or GST was incubated with DPPC liposomes at pH 7.4. Released FFA were quantified after 2 h incubation at 37 °C. PLA2 from bovine pancreas functioned as a positive control. GST and GST–SseJS151V were used as negative controls. The value of FFA following incubation with GST alone was subtracted from values obtained after incubation of DPPC with GST–SseJ or GST–SseJS151V. These data represent the mean±sd derived from three independent experiments performed in triplicate.

To analyse the enzymic activity of SseJ *in vitro*, SseJ and SseJS151V were purified as GST fusion proteins (Fig. 1a[Fig f1]) and incubated with various substrates to test for esterase and PLA activity. As positive controls, porcine liver esterase was used to hydrolyse PNPB (Fig. 1b[Fig f1]) and commercially available PLA2 was used to hydrolyse DPPC in liposomes (Fig. 1c[Fig f1]). No enzymic activity was detected at pH 7.4 when GST–SseJ was incubated with PNPB (Fig. 1b[Fig f1]) or DPPC liposomes (Fig. 1c[Fig f1]). Liposomes consisting of equimolar amounts of cholesterol and DPPC were also used to test whether SseJ displays acyltransferase activity *in vitro*, but no enzymic activity was detected (data not shown). To test whether the absence of detectable activity was due to the 25 kDa GST tag at the N terminus of SseJ, the GST tag was cleaved from the purified protein, but no activity was detected (data not shown). SseJ and SseJS151V were also purified as polyhistidine fusion proteins, but neither had detectable enzymic activity under the assay conditions used (data not shown).

### SseJ displays PLA activity when expressed in HeLa cells

Expression of SseJ following transfection of HeLa cells leads to the formation of globular membranous compartments (GMCs), which are dependent on the catalytic activity of SseJ (Ruiz-Albert *et al.*, 2002). As enzymic activity of recombinant SseJ purified from *E. coli* was undetectable, we investigated its biochemical activity after expression in HeLa cells. HeLa cells were transfected with vectors expressing myc-SseJ or catalytically inactive myc-SseJS151V. Mock-transfected HeLa cells were used as a negative control. Following transfection, HeLa cell lysates were incubated with DPPC liposomes at pH 7.4, 37 °C for 2 h. Released FFA were then quantified. HeLa cell lysate containing myc-SseJ led to the release of more than twice as much FFA as lysate from mock-transfected cells or lysate containing myc-SseJS151V (89.5±11.7 nmol FFA versus 34.0±10.5 nmol FFA and 34.4±6.8 nmol FFA, respectively). This indicates that SseJ possesses PLA activity, which is dependent on the catalytic S in position 151 in the context of HeLa cell lysate.

### Recombinant SseJ displays PLA activity in the presence of HeLa cell extract

**Fig. 2. f2:**
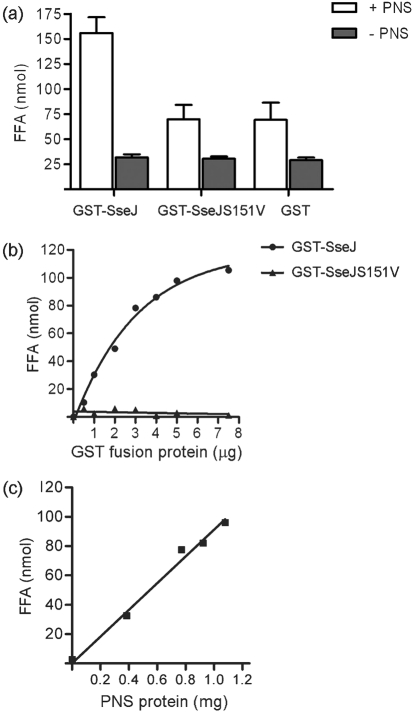
(a) Phospholipase activity of SseJ requires the presence of a eukaryotic factor. GST–SseJ, GST–SseJS151V or GST was incubated with DPPC liposomes in the presence or absence of HeLa cell PNS for 2 h at 37 °C, pH 7.4, before released FFA were quantified. Data represent mean±sd derived from three independent experiments performed in triplicate. (b) Phospholipase activity is dependent on SseJ concentration, but limited by the amount of activator. A fixed amount of HeLa cell extract (0.8 mg protein) was incubated with 1–7.5 μg GST–SseJ (•), GST–SseJS151V (▴) or GST for 2 h at 37 °C, pH 7.4, before FFA were quantified. Values of FFA following incubation with GST were subtracted from values obtained by incubation of DPPC with equivalent amounts of GST–SseJ or GST–SseJS151V. These data represent the mean derived from two independent experiments performed in triplicate. (c) Phospholipase activity as a function of PNS protein amounts. GST–SseJ (5 μg) (▪) or GST was incubated with a range of concentrations of HeLa cell PNS from 0 to 1.1 mg protein at pH 7.4. Released FFA were quantified after 2 h incubation at 37 °C. Values obtained with GST alone were subtracted from values obtained with GST–SseJ. These data represent the mean derived from two independent experiments performed in triplicate.

Since we were able to show phospholipase activity of SseJ following its expression in HeLa cells, but not following its purification after expression in *E. coli*, we hypothesized that it might require a eukaryotic factor for activity. To test this, the activity of GST–SseJ purified from *E. coli* was monitored in the presence of HeLa cell PNS and DPPC liposomes. Incubation of GST–SseJ with DPPC liposomes together with HeLa cell PNS led to the release of 155.9±15.8 nmol FFA compared to 69.7±14.4 nmol and 69.1±17.3 nmol FFA detected after incubation with GST–SseJS151V and purified GST, respectively (Fig. 2a[Fig f2]). In the absence of PNS, FFA was detected at similar low levels following incubation of DPPC liposomes with GST–SseJ, GST–SseJS151V or GST (Fig. 2a[Fig f2]). These results show that a factor present in HeLa cell extract is required for PLA activity of SseJ. Incubation of increasing amounts of GST–SseJ with a fixed amount of DPPC liposomes and a fixed amount of HeLa cell extract led to an increase in the amount of released FFA up to approximately 5 μg GST–SseJ, after which production of FFA plateaued (Fig. 2b[Fig f2]). To determine whether the substrate concentration or activating factors were limiting under these conditions, 5 μg GST–SseJ and DPPC liposomes were incubated with an increasing amount of eukaryotic cell extract, which led to a linear increase in released FFA (Fig. 2c[Fig f2]). This shows that HeLa cell-derived factor(s) limit the reaction under the assay conditions used.

### SseJ does not display lysophospholipase activity

**Fig. 3. f3:**
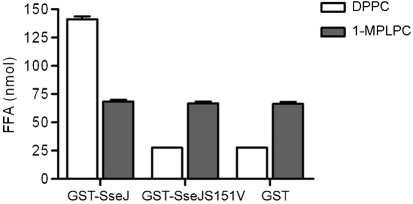
SseJ does not show lysophospholipase activity *in vitro*. GST–SseJ, GST–SseJS151V or GST was incubated with DPPC or 1-MPLPC liposomes at pH 7.4 in the presence of HeLa cell lysate for 2 h at 37 °C before FFA were quantified. Lysis of DPPC by GST–SseJ served as a positive control. GST and GST–SseJS151V were used as negative controls for both DPPC and 1-MPLPC lysis. These results represent the mean±sd for each sample (in triplicate), and are representative of two independent experiments.

To analyse whether SseJ hydrolyses lysophospholipids, GST–SseJ, GST–SseJS151V or GST was incubated with 1-MPLPC micelles at 37 °C, pH 7.4 for 2 h in the presence of HeLa cell PNS. In contrast to incubation of GST–SseJ with DPPC liposomes, the amount of FFA after incubation of GST–SseJ with 1-MPLPC micelles did not significantly differ from the amount of FFA detected in the presence of GST–SseJS151V or GST (Fig. 3). Therefore, SseJ does not exhibit lysophospholipase activity at pH 7.4 in the presence of HeLa cell PNS using 1-MPLPC micelles as a substrate.

### HeLa cell extract-activated SseJ displays GCAT activity

**Fig. 4. f4:**
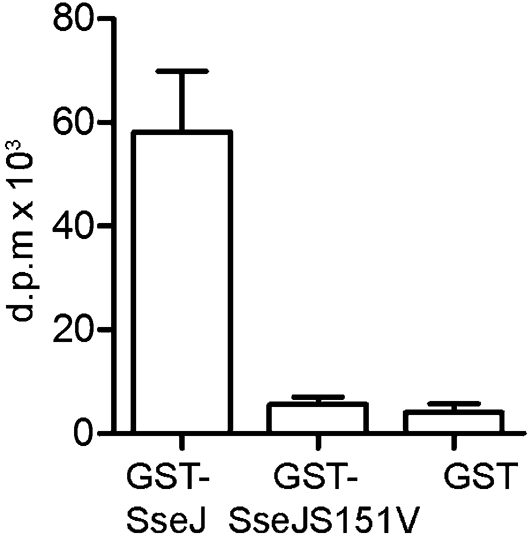
SseJ displays GCAT activity in the presence of HeLa cell PNS. GST–SseJ, GST–SseJS151V or GST was incubated with DPPC/cholesterol (1 : 1, mol/mol) liposomes containing [^3^H]cholesterol in the presence of HeLa cell PNS at pH 7.4. After 2 h incubation, lipids were extracted and separated by TLC. Levels of [^3^H]CEs were measured by detection of d.p.m. in the CE fraction. These results represent the mean±sd for each sample (in triplicate) and are representative of three independent experiments.

To test whether SseJ can carry out acyl transfer in the presence of eukaryotic activator, GST–SseJ, GST–SseJS151V or GST was incubated in the presence of HeLa cell PNS with liposomes comprising an equimolar mix of DPPC and partially ^3^H-labelled cholesterol. The formation of ^3^H-labelled CE was measured after lipid extraction and separation via TLC. In the presence of GST–SseJ and HeLa cell PNS, 58 214.8±1162.6 d.p.m. were detected in the CE fraction, while GST–SseJS151V and GST produced 5543.7±1418.5 d.p.m. and 4011.6±1710.3 d.p.m., respectively, demonstrating that SseJ possesses GCAT activity in the presence of HeLa cell extract (Fig. 4).

### Analysis of the SseJ-activating factor(s)

**Fig. 5. f5:**
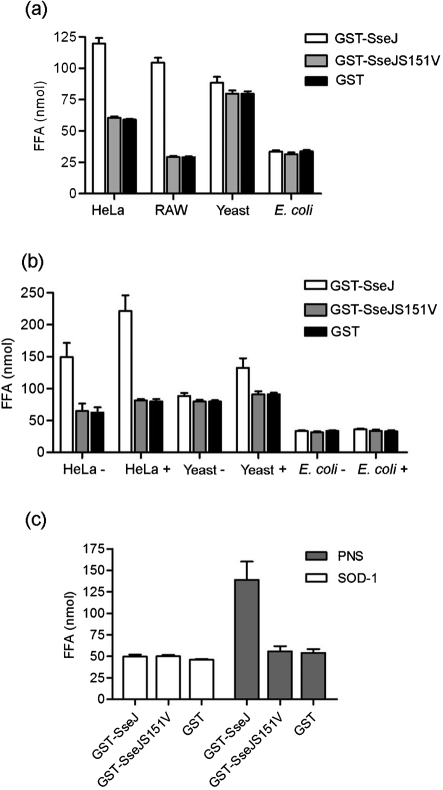
(a) GST–SseJ, GST–SseJS151V or GST was incubated with DPPC liposomes at 37 °C, pH 7.4, in the presence of cell lysates extracted from HeLa cells, RAW macrophages, *Sacc. cerevisiae* (Yeast) or *E. coli*. Released FFA were quantified after 2 h incubation. (b) GST–SseJ, GST–SseJS151V or GST was incubated with HeLa cell, *E. coli* or *Sacc. cerevisiae* extract for 1 h at 37 or 30 °C (*Sacc. cerevisiae*), respectively, before addition of DPPC liposomes. FFA were quantified after further incubation at 37 °C for 2 h (HeLa+/Yeast+/*E. coli*+). Released FFA were compared to FFA released in identical assays without pre-incubation of GST fusion proteins (HeLa−/Yeast−/*E. coli*−). (c) GST–SseJ, GST–SseJS151V or GST (5 μg) was pre-incubated with 20 μg SOD-1 for 1 h at 37 °C before addition of DPPC. In a final volume of 200 μl, FFA were quantified after further incubation for 2 h at 37 °C. Results shown in (a–c) represent the mean±sd for each sample (in triplicate) and are representative of two independent experiments.

HeLa cell extract was required for PLA and GCAT activity of SseJ *in vitro*. In addition to HeLa cell extract, mouse macrophage RAW 264.7 cell extract contained factor(s) that activated SseJ (Fig. 5a[Fig f5]). Soluble extracts of *E. coli*, *S*. Typhimurium or *Sacc. cerevisiae* did not activate SseJ (Fig. 5a[Fig f5] and data not shown). Interestingly, the activity of GST–SseJ was detectable when GST–SseJ was pre-incubated with *Sacc. cerevisiae* extract at 30 °C for 1 h before addition of DPPC liposomes and incubation for 2 h at 37 °C (FFA released without pre-incubation, 88.38±4.84 nmol; FFA released after pre-incubation, 132.48±14.9 nmol FFA) indicating that *Sacc. cerevisiae* extract also contained SseJ-activating factor(s) (Fig. 5b[Fig f5]). Similarly, incubation of GST–SseJ with HeLa cell extract for 1 h at 37 °C prior to addition of DPPC liposomes also led to an increase in released FFA compared to FFA released by GST–SseJ that was incubated with activator and substrate simultaneously (221.50±24.69 nmol FFA versus 149.48±22.12 nmol) (Fig. 5b[Fig f5]). However, pre-incubation of GST–SseJ with *E. coli* soluble extract did not result in detectable PLA activity of GST–SseJ. We conclude that SseJ was activated by factor(s) present in *Sacc. cerevisiae* extract only after pre-incubation, suggesting that the concentration of activator might be lower in yeast extract than in HeLa or RAW cell extract.

**Fig. 6. f6:**
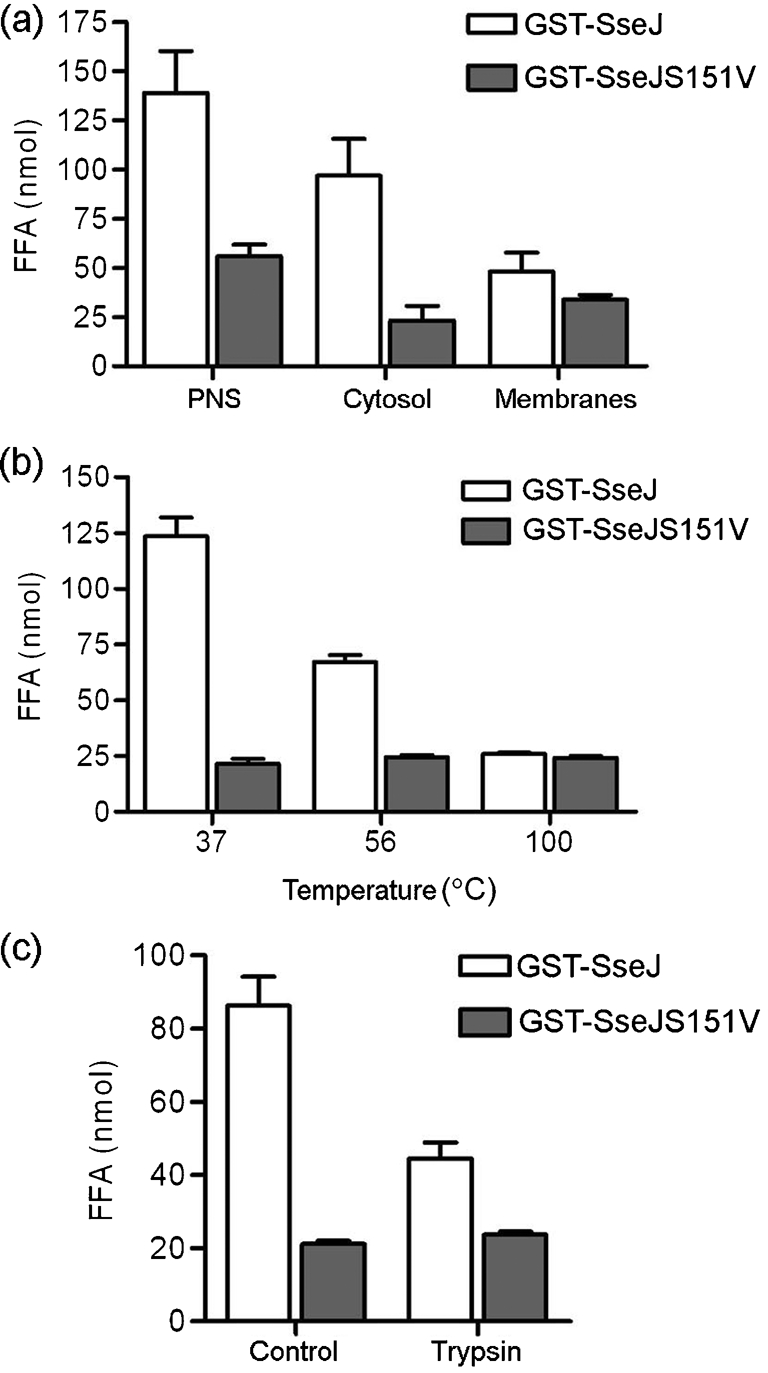
GST–SseJ, GST–SseJS151V or GST was incubated with DPPC liposomes in the presence of HeLa cell extract. Conditions of cell extract preparation are described below. Data represent the amount of FFA quantified after incubation of GST fusion proteins with DPPC liposomes and cell extract for 2 h at 37 °C. (a) PNS of HeLa cells was fractionated into cytosolic and membrane fractions by ultracentrifugation. Equivalent proportions of cytosol and membrane fraction were added to the assay. Data shown represent mean±sd derived from three independent experiments. (b) HeLa cell cytosol was subjected to 37 °C for 30 min, 56 °C for 30 min or 100 °C for 5 min before addition to the phospholipase assay. Data represent mean±sd derived from two independent experiments performed in triplicate. (c) HeLa cell cytosol was pre-treated with trypsin for 15 min. The pre-treated cytosol was added to the phospholipase assay after addition of the protease inhibitor aprotinin. In the control, protease inhibitor was added to the cytosolic fraction before addition of trypsin. The results represent the mean±sd for each sample (in triplicate) and are representative of three independent experiments.

Several phospholipases are known to require divalent cations, such as Ca^2+^ (Clark *et al.*, 1991; Dessen *et al.*, 1999; Reynolds *et al.*, 1993). The addition of 10 mM CaCl_2_, MgCl_2_ or ZnCl_2_ did not activate SseJ phospholipase activity *in vitro* (data not shown). Fractionation of HeLa cell PNS into cytosolic and membrane fractions prior to addition to the assay demonstrated that the activator was mainly retained in the cytosolic fraction (Fig. 6a[Fig f6]). Incubating HeLa cell cytosol at 56 °C decreased its ability to activate SseJ and treatment of cytosol at 100 °C led to a complete loss of activating ability (Fig. 6b[Fig f6]). When cytosol was pre-treated with trypsin before incubation with SseJ, the activating ability of the extract was reduced (Fig. 6c[Fig f6]). After subjecting cytosol to size-exclusion filtration with 100 kDa cut-off, the activating factor was retained in the >100 kDa fraction (data not shown). This shows that the activating activity is likely to be proteinaceous and to have a molecular mass over 100 kDa or to be present in a complex with a mass of >100 kDa.

To analyse whether cleavage or other covalent modification of SseJ is responsible for its activation, macrophages were infected with strains of *S*. Typhimurium expressing double HA-tagged SseJ (SseJ–2HA) for 14 h. The electrophoretic mobility of translocated SseJ was compared with SseJ produced by *S*. Typhimurium grown *in vitro*. No differences in electrophoretic mobility were detected (data not shown).

Superoxide dismutase (SOD-1) is known to be required for the activity of ExoU, a T3SS effector with phospholipase activity expressed by *Pseudomonas aeruginosa* (Sato *et al.*, 2006). GST–SseJ, GST–SseJS151V or GST was therefore incubated with bovine SOD-1 in excess for 1 h at 37 °C prior to addition of DPPC liposomes and further incubation for 2 h at 37 °C, pH 7.4; however, no activity of GST–SseJ was detected (Fig. 5c[Fig f5]). Therefore, SseJ is likely to be activated by a novel eukaryotic proteinaceous factor.

## DISCUSSION

In this work we have analysed the biochemical activity of SseJ, a *Salmonella* SPI-2 T3SS effector protein. We found that both the PLA activity and the GCAT activity of SseJ need to be activated by a factor or factors present in eukaryotic cells. We have not yet established the identity of the activator but it is likely to be proteinaceous. There is a formal possibility that a dormant eukaryotic enzyme could be activated (directly or indirectly) by SseJ. This can only be ruled out (or confirmed) conclusively by identification of the activator itself. However, a dormant eukaryotic enzyme seems highly unlikely, given the amino acid sequence similarity between SseJ and other GDSL lipases and GCAT of *A. hydrophila*. Purified SseJ has been reported to possess deacylase activity on PNPB (Ohlson *et al.*, 2005) and GCAT activity on phosphatidylcholine/cholesterol liposomes (Nawabi *et al.*, 2008). However, we were unable to detect deacylase or GCAT activity of SseJ in the absence of activator using very similar assay conditions to those described elsewhere (Nawabi *et al.*, 2008; Ohlson *et al.*, 2005).

The N-terminal 140 aa of SseJ have similarity to several other SPI-2 effectors and contain a signal for its translocation (Miao & Miller, 2000). The region of SseJ encompassing amino acids 140–408, however, is similar to several members of the GDSL lipase family, including GCAT of *Aeromonas* spp. (26.8 % amino acid identity) (Brumlik & Buckley, 1996) and PlaC of *Legionella pneumophila* (19.5 % amino acid identity), which also possesses GCAT activity (Banerji *et al.*, 2005; Brumlik & Buckley, 1996). The GDSL lipase family is characterized by five conserved blocks of amino acids. The first contains the GDSL motif including the catalytic serine, and the third and fifth contain aspartic acid and histidine residues, respectively; together with the serine in block 1 these constitute the catalytic triad (Akoh *et al.*, 2004). The corresponding residues in SseJ (Table 2) are essential for the function of the protein *in vivo* and for deacylase activity *in vitro* (Ohlson *et al.*, 2005), and we show in this paper that the predicted catalytic serine in block 1 is essential for both PLA and GCAT activity when stimulated by eukaryotic cell extract. It is not clear why Nawabi *et al.* (2008) were able to detect GCAT activity in the absence of the activator while we were not. It is possible that differences in methods of enzyme expression and purification, substrate choice (synthetic DPPC versus natural egg phosphatidylcholine) or lipid extraction could have revealed activity in the absence of eukaryotic activator. Notwithstanding this discrepancy, we have clearly demonstrated that the GCAT activity of SseJ is significantly enhanced after exposure to (a) eukaryotic factor(s).

Both GCAT and PlaC need to be activated by proteases (Banerji *et al.*, 2005; Vipond *et al.*, 1998). GCAT activity is potentiated as a result of proteolytic processing by AspA, the major secreted serine protease of *Aeromonas salmonicida* (Hilton *et al.*, 1990; Vipond *et al.*, 1998). Pro-GCAT (37 kDa) is cleaved at two sites, resulting in three GCAT fragments, two of which are connected via a disulfide bond (33 kDa), so that only a very small fragment of pro-GCAT is lost (Vipond *et al.*, 1998). Both pro- and processed GCAT possess activity *in vitro*, but only processed GCAT can penetrate lipid monolayers at surface pressures equivalent to those of natural membranes (>30 mN m^−1^) (Hilton *et al.*, 1990; Hilton & Buckley, 1991). Activation of PlaC is dependent on the zinc metalloprotease ProA, although it is not clear whether this effect is direct or indirect (Banerji *et al.*, 2005). Despite these two precedents, it seems unlikely that SseJ is activated by proteolytic cleavage, since several protease inhibitors were added to cell extracts prior to incubation with SseJ and no size difference between intrabacterial and translocated SseJ was detected by Western blotting. Another difference from GCAT and PlaC is that SseJ requires (a) eukaryotic factor(s) for its activity. ExoU, a *P. aeruginosa* T3SS effector protein with PLA2 activity (belonging to the patatin-like lipase family), also requires a eukaryotic activator, which has recently been identified as superoxide dismutase (SOD-1) (Sato *et al.*, 2006). However, the mechanism by which SOD-1 activates ExoU has not yet been elucidated. Purified bovine SOD-1 did not activate SseJ in *vitro*, and it therefore seems likely that SseJ is activated by a unique mechanism. Attempts to identify the eukaryotic activator(s) are underway and this will provide further insight into the mechanism of activation of SseJ.

It is interesting to consider how the GCAT activity of SseJ might influence the biology of the SCV. SseJ is translocated by the SPI-2 T3SS and localizes to the cytosolic face of the vacuole and Sifs (Freeman *et al.*, 2003), tubular extensions of the SCV which form along microtubules, and which are particularly noticeable in epithelial cells (Garcia-del Portillo *et al.*, 1993). Therefore, SseJ is likely to act on phospholipids, present in the SCV membrane and Sifs, transferring acyl chains to cholesterol, which appears by microscopy to be particularly abundant in the SCV membrane (Catron *et al.*, 2002). Although the resolution of light microscopy does not allow one to conclude that cholesterol is present in the SCV membrane itself, immunolabelling of intra-vacuolar *Salmonella* after exposure of the SCV to saponin (a cholesterol-dependent membrane-permeabilizing reagent) indicates that cholesterol is present in the SCV membrane. The function of SseJ is linked to that of SifA, an SPI-2 T3SS effector whose function has been clarified in recent years. Following translocation and localization to the SCV membrane and Sifs, SifA is prenylated and anchored in the SCV membrane of SCVs and Sifs (Reinicke *et al.*, 2005). SifA binds to SKIP, a host cell protein that prevents the microtubule motor kinesin from being recruited to the SCV. *sifA* mutants fail to induce the formation of Sifs (Stein *et al.*, 1996) and gradually lose their vacuolar membranes (Beuzon *et al.*, 2000) in a kinesin-dependent manner (Guignot *et al.*, 2004).

Two intracellular phenotypes have been described that result from mutation of *sseJ*. First, the loss of vacuolar membrane around *sifA* mutants requires the activity of SseJ (Ruiz-Albert *et al.*, 2002), and second, deletion of *sseJ* induces the formation of more Sifs per cell, suggesting that SseJ inhibits Sif formation (Birmingham *et al.*, 2005). Therefore, SseJ appears to oppose the activity of SifA. If the GCAT activity of SseJ in the SCV membrane esterifies cholesterol and removes it from the SCV to lipid droplets (Nawabi *et al.*, 2008), its absence could increase membrane rigidity, facilitating kinesin-mediated rupture of vacuolar membranes around *sifA* mutant bacteria. Membrane association of prenylated Rab proteins is dependent on the cholesterol content of the membrane (Chen *et al.*, 2008; Lebrand *et al.*, 2002). Hence, by regulating the amount of cholesterol in the SCV membrane, SseJ could control the amount of SifA on the SCV. In the *sseJ* mutant, more cholesterol could lead to greater incorporation of SifA into the SCV membrane and thereby to an increased level of Sif formation.

The dynamic properties of the SCV membrane are also likely to be altered by SseJ-mediated deacylation of phospholipids, since membrane curvature is affected by the lipid composition of the phospholipid bilayer (McMahon & Gallop, 2005). Furthermore, GCAT activity could influence host cell signalling pathways by affecting lipid raft composition through its effect on cholesterol and by generating FFA and lysophospholipid.

## Figures and Tables

**Table 1. t1:** Strains and plasmids used in this study

**Strain**	**Description**	**Reference/source**
*Sacc. cerevisiae* AH 109	*Sacc. cerevisiae*	BD Biosciences, Clontech
*E. coli* BL21 (DE3)	*E. coli* BL21 (DE3)	Amersham Biosciences
*S*. Typhimurium 12023	Wild-type *S*. Typhimurium	NTCC (Colindale, UK)
pGEX4T-2	Vector containing gene encoding GST	Amersham Biosciences
pGEX4T2 : : *sseJ*	Vector expressing GST–SseJ	This study
pGEX4T2 : : *sseJ*_mt_	Vector expressing GST–SseJS151V	This study
p*myc* : : *sseJ*	pRK5myc : : *sseJ*	Ruiz-Albert *et al.* (2002)
p*myc* : : *sseJ*_S151V_	pRK5myc : : *sseJ*_S151V_	Ruiz-Albert *et al.* (2002)

**Table 2. t2:** Alignment of several members of the GDSL lipase family The three residues highlighted in bold type (‘S’ in Block I, ‘D’ in Block III and ‘H’ in Block V) represent the catalytic triad conserved in GDSL lipases.The additional spaces have been inserted to maintain the correct sequence alignment.

**Organism**	**Accession number**	**Name and function**	Δ*	**Block I**	Δ*	**Block II**	Δ*	**Block III**	Δ*	**Block IV**	Δ*	**Block V**	Δ*
*A. salmonicida*	AAG09804	GCAT	27	IVMFGD**S**LSDT G	38	LT IANEAEGGPT	37	VILWVGAN**D**YL	26	NGAKEI LL FNLPD	128	FWDQV**H**PT	25
*S.* Typhimurium	AAG02230	SseJ, GCAT	144	LVFFGD**S**LSDSLG	37	KEMLNFAEGGST	31	AIFL LGAN**D**YM	23	GGVNNVLVMGIPD	93	FN DLV**H**PT	22
*L. pneumophila*	AY745197	PlaC, GCAT	30	IVVFGD**S**LSDNG	108	EVYLNKAFGGSW	53	YFIYSGS N**D**YI	34	AGARRFVIMGIPH	123	FWDE I**H**PT	30

*Δ Values in the table headings represent the number of amino acids between adjacent conserved blocks.

## Abbreviations

- CE, cholesterol ester
- DPPC, 1,2-dipalmitoylphosphatidylcholine
- FFA, free fatty acids
- GCAT, glycerophospholipid : , cholesterol acyltransferase
- GST, glutathione-*S*-transferase
- 1-MPLPC, 1-monopalmitoyllysophosphatidylcholine
- PLA, phospholipase A
- PLA2, phospholipase A2
- PNPB, *para*-nitrophenyl butyrate
- PNS, postnuclear supernatant
- SCV, *Salmonella*-containing vacuole
- SPI-2, *Salmonella* pathogenicity island 2
- T3SS, type III secretion system
